# Pan-cancer analysis of Sushi domain-containing protein 4 (SUSD4) and validated in colorectal cancer

**DOI:** 10.18632/aging.205712

**Published:** 2024-04-04

**Authors:** Yuchen Zhong, Chaojing Zheng, Weiyuan Zhang, Hongyu Wu, Qian Zhang, Dechuan Li, Haixing Ju, Haiyang Feng, Yinbo Chen, Yongtian Fan, Weiping Chen, Meng Wang, Guiyu Wang

**Affiliations:** 1 Zhejiang Cancer Hospital, Hangzhou Institute of Medicine (HIM), Chinese Academy of Sciences, Hangzhou 310022, Zhejiang, China; 2 Department of Colorectal Cancer Surgery, The Second Affiliated Hospital of Harbin Medical University, Harbin 150000, Heilongjiang, China

**Keywords:** complement system, systemic pan-cancer analysis, SUSD4, immune infiltration of tumors, prognosis

## Abstract

Sushi domain-containing protein 4 (SUSD4) is a complement regulatory protein whose primary function is to inhibit the complement system, and it is involved in immune regulation. The role of SUSD4 in cancer progression has largely remained elusive. SUSD4 was studied across a variety of cancer types in this study. According to the results, there is an association between the expression level of SUSD4 and prognosis in multiple types of cancer. Further analysis demonstrated that SUSD4 expression level was related to immune cell infiltration, immune-related genes, tumor heterogeneity, and multiple cancer pathways. Additionally, we validated the function of SUSD4 in colorectal cancer cell lines and found that knockdown of SUSD4 inhibited cell growth and impacted the JAK/STAT pathway. By characterizing drug sensitivity in organoids, we found that the expression of SUSD4 showed a positive correlation trend with IC50 of Selumetinib, YK-4-279, and Piperlongumine. In conclusion, SUSD4 is a valuable prognostic indicator for diverse types of cancer, and it has the potential to be a target for cancer therapy.

## INTRODUCTION

Complement fragments play a pivotal role in the defense against foreign pathogens, representing a cornerstone of the innate immune system [1]. Moreover, the activation of the complement system contributes significantly to anticancer defense mechanisms, with Sushi domain-containing protein 4 (SUSD4) emerging as a notable regulatory player within this context. Structurally akin to the membrane complement inhibitor CD46, the immunological significance of SUSD4 warrants particular scrutiny, given the pivotal role of its analogous protein, CD46, in T cell functions [2]. Additionally, CD46 is closely associated with IL-10 and TH1 cells, suggesting that SUSD4 may have a similar function to CD46 [3, 4]. Emelie et al. demonstrated that downregulation of SUSD4 in breast malignancy cells was related to a poor prognosis, and knockdown of SUSD4 in breast malignancy cell lines reduced cell migration and invasion [5, 6].

Nevertheless, the exploration of SUSD4’s involvement in tumor immunity and prognostic implications across various cancer types remains limited in prior research efforts. Adopting a systematic pan-cancer approach, the present investigation aims to elucidate the multifaceted dynamics of SUSD4 expression levels vis-à-vis cancer prognosis. Leveraging data extraction from diverse databases, coupled with bioinformatics analyses, we endeavor to delineate the intricate interplay between SUSD4 expression and the immune microenvironment, along with its ramifications on immune-related genes. Additionally, we employ a pharmacological lens to identify potential therapeutics targeting SUSD4.

In sum, our study provides pioneering insights into the prognostic and immunological significance of SUSD4, offering a valuable foundation for the exploration of novel immunotherapeutic targets and informing the development of more efficacious treatment modalities by aiding clinicians in optimizing therapeutic strategies.

## RESULTS

### SUSD4 expression level in normal and tumor samples

**Figure 1:**
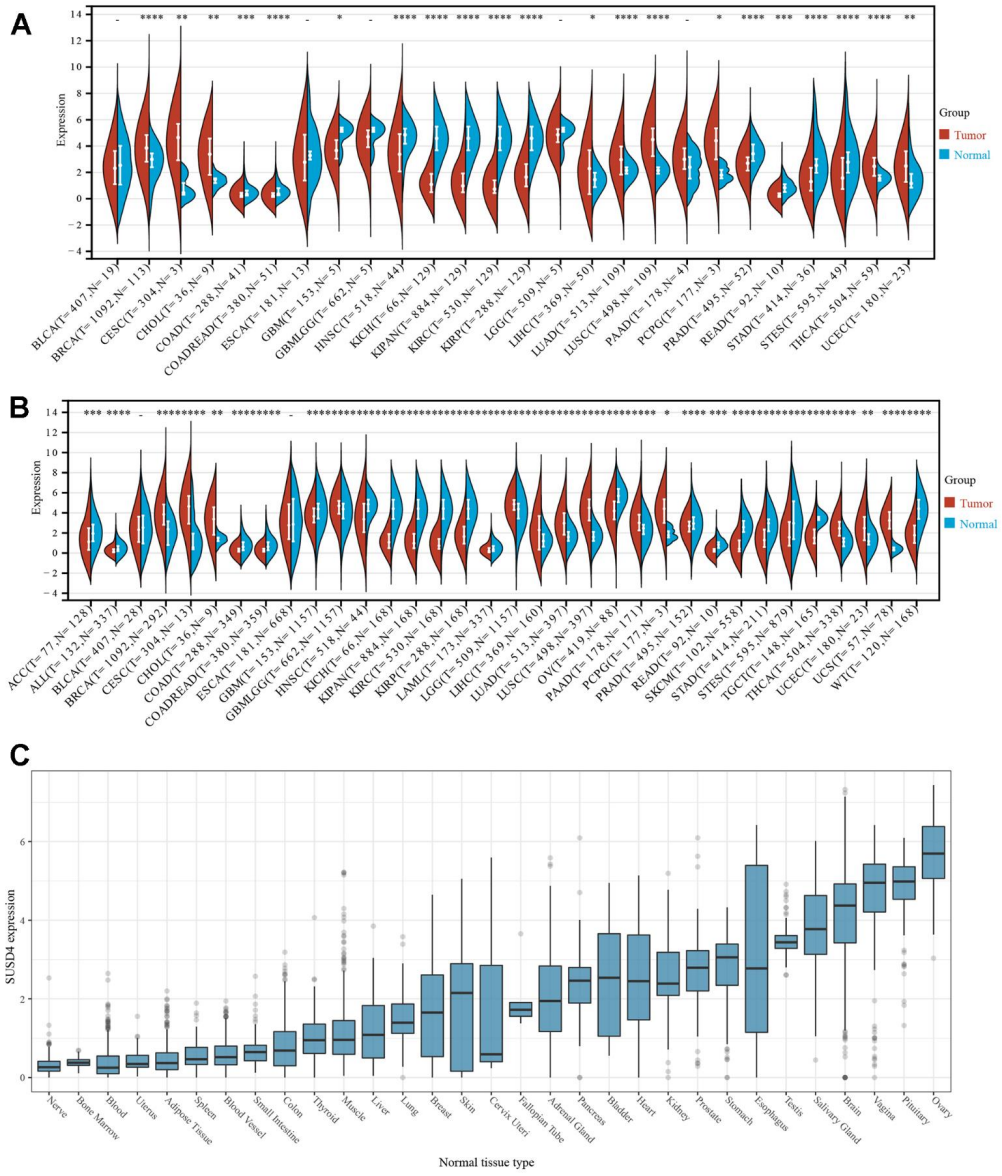
**The expression of SUSD4 in pan-cancer.** (**A**) Pan-cancer analysis of SUSD4 expression across cancers from TCGA. (**B**) Expression profiles across GTEx tissues and TCGA. (**C**) Expression of SUSD4 in cell lines from CCLE dataset. *p < 0.05, **p < 0.01, ***p < 0.001, and ****p < 0.0001. Abbreviation of cancers are available in the Supplementary Table 1.

SUSD4 expression levels in normal and tumor samples were compared using the TCGA database (Figure 1A). It was observed that SUSD4 expression was significantly upregulated in 9 tumors, such as BRCA, CESC, CHOL, LIHC, LUAD, LUSC, PCPD, THCA, and UCEC. Conversely, significant downregulation of SUSD4 expression was observed in COAD, COREAD, GBM, HNSC, KICH, KIPAN, KIRP, KIRC, PRAD, STAD, STES, and READ. Comparison of SUSD4 expression levels in normal and tumor samples was also performed using data obtained from the GTEx database (Figure 1B). Significant upregulation of SUSD4 expression was noted in 12 tumors, including BRCA, CESC, CHOL, LGG, LIHC, LUAD, LUSC, PAAD, PCPG, THCA, UCEC, and UCS. In the other 13 tumors (ACC, COREAD, GBM, HNSC, KICH, KIRC, KIPC, LAML, OV, PRAD, SKCM, STAD, and TGCT), SUSD4 expression was downregulated.

We obtained the expression matrix of SUSD4 in various cancer cell lines through the CCLE database and found that the expression level of SUSD4 was highest in breast cancer, while it was lowest in lymphoma, rhabdoid, and myeloma (Figure 1C). However, overall, the expression level of SUSD4 is not high across multiple cell lines.

### Survival and prognosis analyses

**Figure 2:**
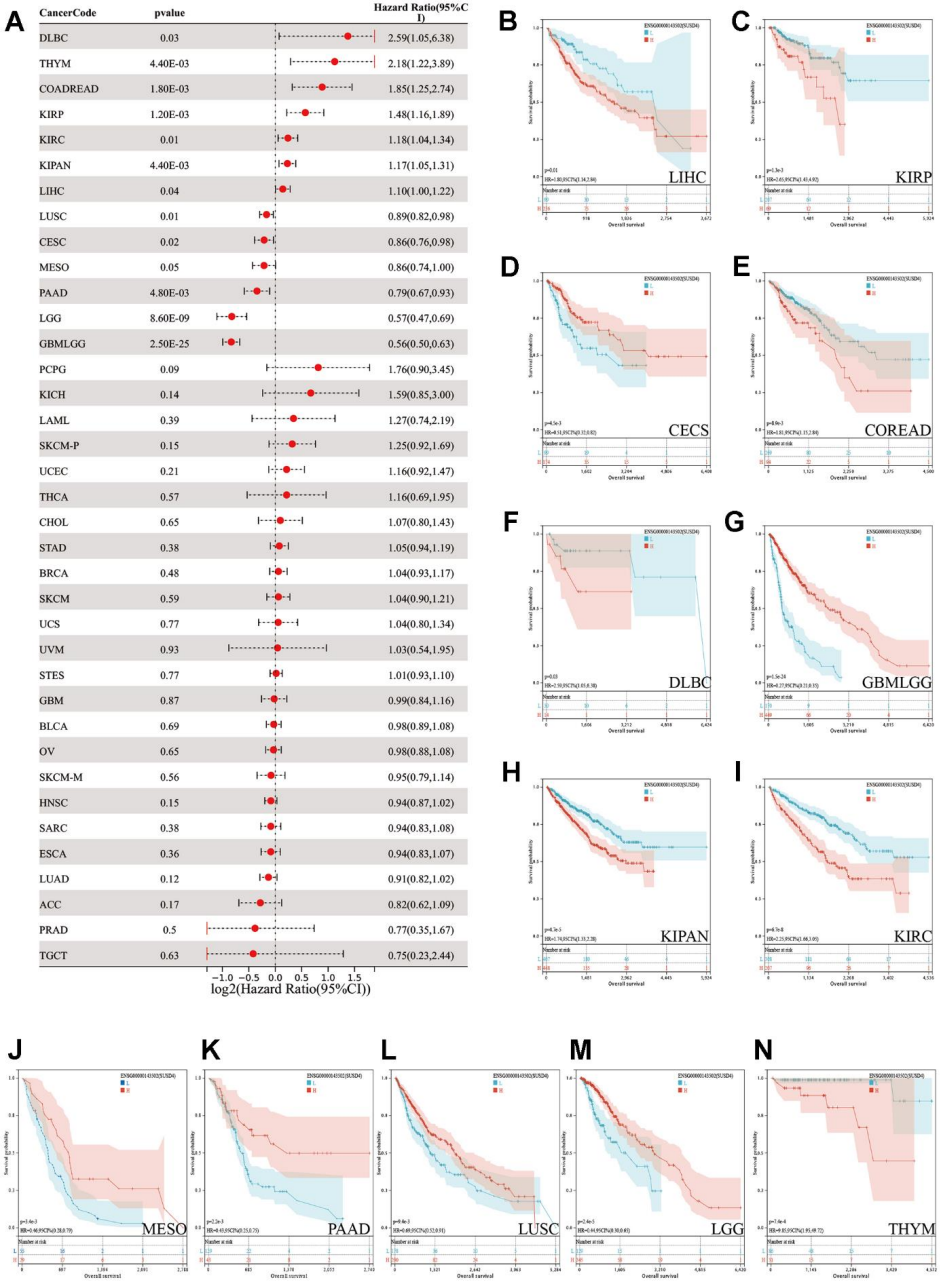
**Pan-cancer survival analysis of SUSD4.** (**A**) Forest plot of the pan-cancer survival analysis. (**B**–**N**) The Kaplan-Meier plot of TCGA pan-cancer survival profiles with significant differences. The red and blue line represents high and low SUSD4 expression group respectively. HR, hazard radio. CI, confidence interval.

We evaluated the prognostic significance of SUSD4 expression levels in various tumor types using the log-rank test. Our analysis revealed notably high SUSD4 expression in KIRP (p=1.2e-3, hazard ratio (HR)=1.48 (1.16,1.89)), KIPAN (p=4.4e-3, HR=1.17 (1.05,1.31)), KIRC (p=0.01, HR=1.18 (1.04, 1.34)), COADREAD (p=1.8e-3, HR=1.85 (1.25, 2.74)), THYM (p=4.4e-3, HR=2.18 (1.22, 3.89)), LIHC (p=0.04, HR=1.10 (1.00,1.22)), and DLBC (p=0.03, HR=2.59 (1.05,6.38)), which was associated with shorter overall survival (OS). In contrast, low SUSD4 expression levels were observed in five tumors (GBMLGG (p=2.5e-25, HR=0.56(0.50,0.63), LGG (p=8.6e-9, HR=0.57 (0.47,0.69), CESC (p=0.02, HR=0.86 (0.76,0.98), LUSC (p=0.01, HR=0.89 (0.82,0.98), and PAAD (p=4.8e-3, HR=0.79 (0.67,0.93)), which was accompanied by a poor prognosis (Figure 2A). Interestingly, high expression of SUSD4 in LGG is a prognostic protective factor, while in GBM, the expression level of SUSD4 is not significantly associated with survival.

**Figure 3:**
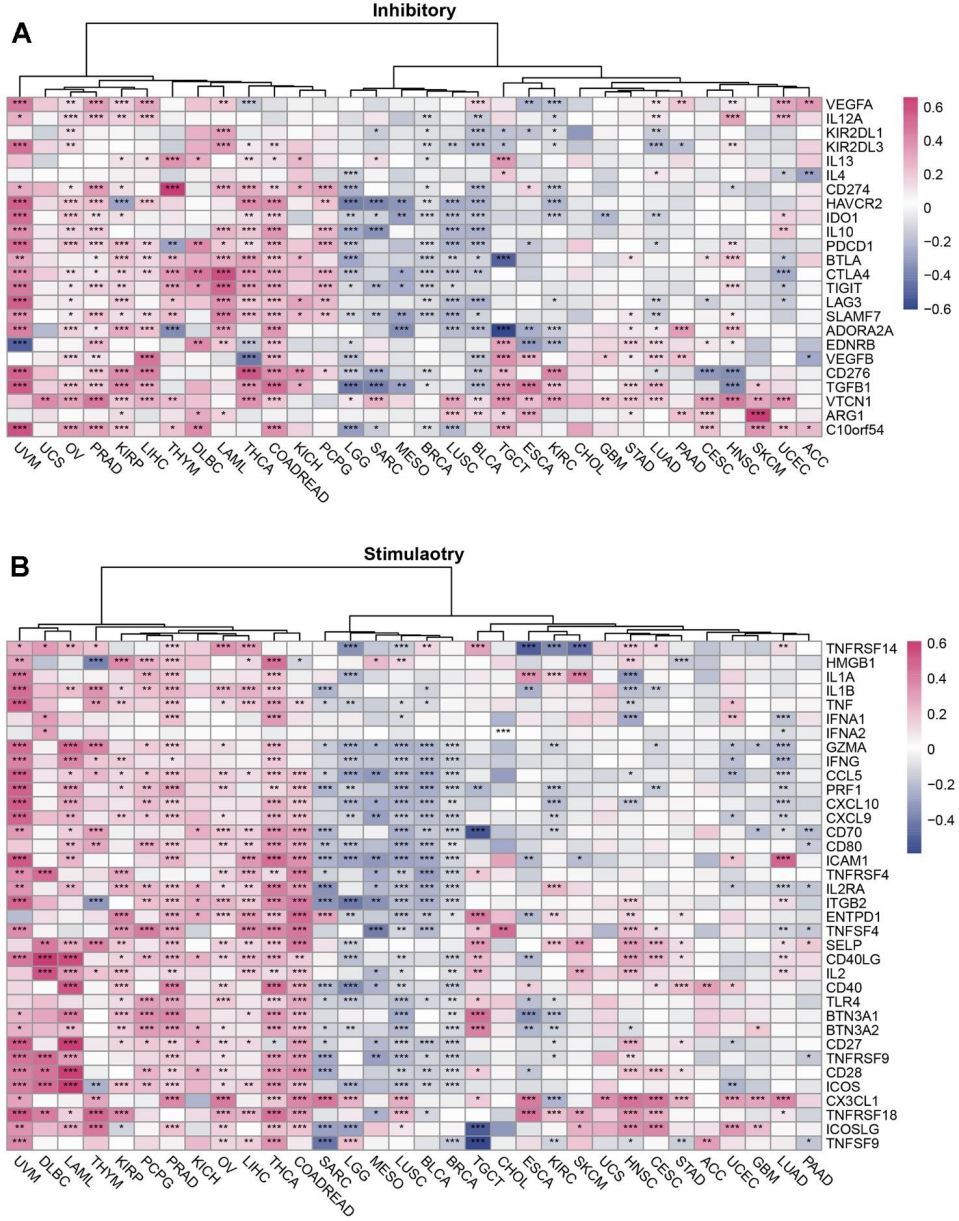
**The relationship between SUSD4 and immune checkpoint genes in pan-cancer.** Correlation Heatmap of SUSD4 with Two Types of Immune Checkpoint Pathway Genes: (**A**) Inhibitory and (**B**) Stimulatory. *p < 0.05, **p < 0.01, ***p < 0.001. P-values were adjusted by false discovery rate (FDR) method.

Based on the optimal cutoff value, patients were divided into two groups, and the prognostic differences were further analyzed using the ‘survfit’ function of the ‘survival’ R package. Differences in prognostic significance were assessed using the log-rank test, and the Kaplan-Meier (KM) survival curves were plotted (Figure 3B–3N). Overall, SUSD4 exhibits a dichotomous relationship with prognosis in different types of cancer.

### Immunological genes and SUSD4

**Figure 4:**
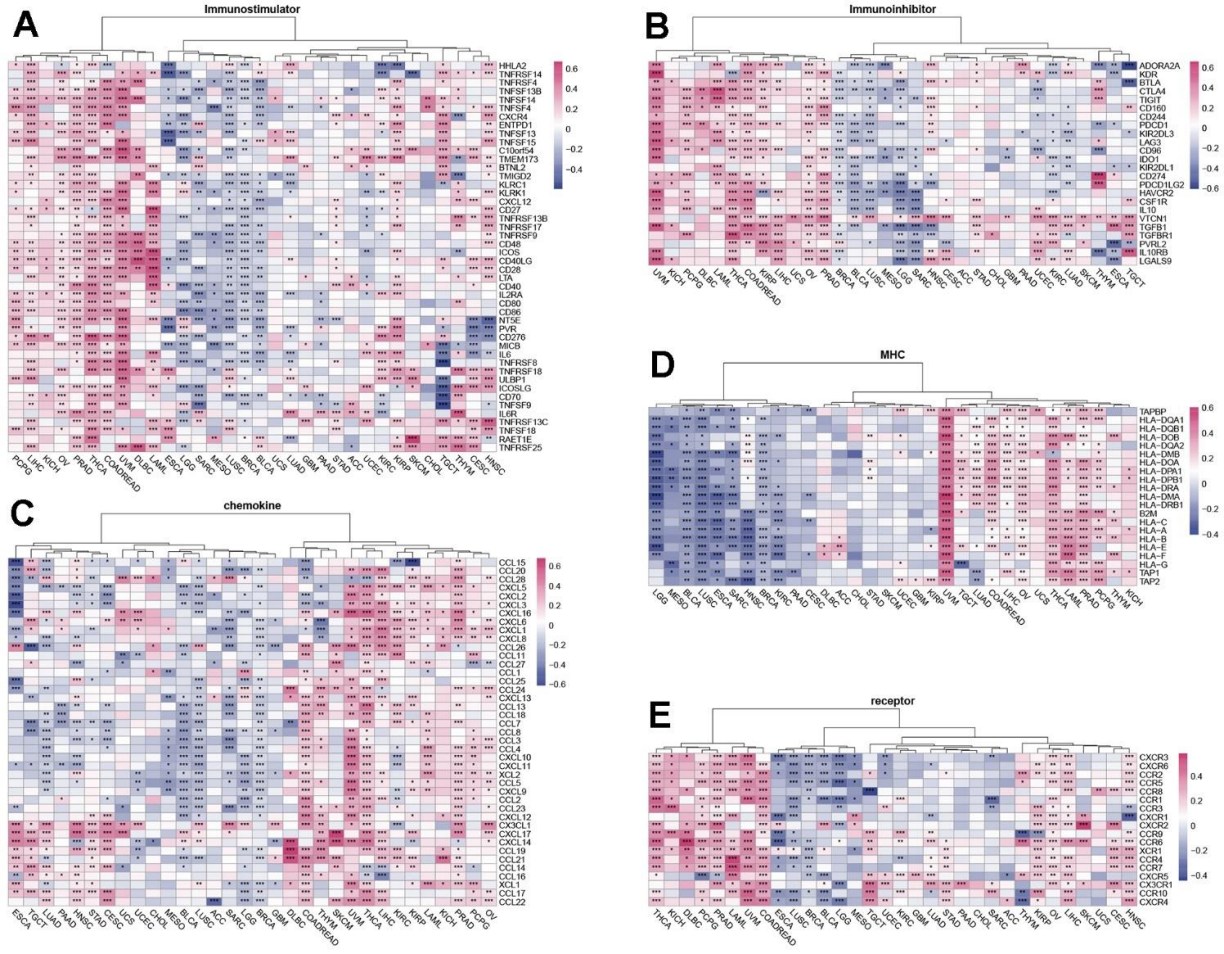
**Relationship of SUSD4 expression with immune-related genes.** The correlation of SUSD4 expression level with immunostimulatory (**A**) and immunoinhibitory genes (**B**). Correlation of chemokine genes (**C**), MHC genes (**D**) and receptor genes (**E**) and SUSD4. *p < 0.05, **p < 0.01, ***p < 0.001. P-values were adjusted by false discovery rate (FDR) method.

SUSD4, a complement regulator, is associated with a variety of immune-related genes. Therefore, a correlation was found between SUSD4 expression levels and the expression levels of multiple immune-related genes in different tumor tissues (Figures 3, 4). The correlation of SUSD4 expression level with expression levels of immunoinhibitory and immunostimulatory genes is shown using a heatmap in Figure 3A, 3B.

Among the immunoinhibitory genes, VTCN1 expression level was positively correlated with SUSD4 expression level in many types of cancer (Figure 3A). However, SUSD4 expression level was negatively correlated with the expression levels of LGG, SARC, BRCA, LUSC, and BLCA. Conversely, SUSD4 expression level was positively correlated with the expression levels of KIRP, COADREAD, PRAD, OV, THCA, HNSC, UVM, THYM, and LIHC. Interestingly, in CHOL, there was no statistically significant correlation between the expression level of SUSD4 and inhibitory-related genes. Similarly, in UCS, only the expression levels of VTCN1 and SUSD4 showed a positive correlation. Additionally, in cancers with poor prognosis and high SUSD4 expression (including DLBC, THYM, COADREAD, KIRP, LIHC), a positive correlation trend was observed with inhibitory-related genes.

Figure 3B shows the correlation between the expression levels of stimulatory genes and SUSD4. We found a positive correlation in UVM, DLBC, LAML, THYM, KIRP, PCPG, PRAD, THCA, and COADREAD. Conversely, there is a general trend of a negative correlation in SARC, LGG, MESO, LUSC, BLCA, and BRCA. The correlation is relatively weak in other types.

The use of immune checkpoint inhibitor-based immunotherapy is exponentially increasing in the treatment of patients with advanced cancer. It was revealed that SUSD4 expression level was positively correlated with immune checkpoints by analyzing the correlation of SUSD4 expression level with the expression levels of immune checkpoint-related genes in PCPG, LIHC, OV, PRAD, THCA, COADREAD, UVM, DLBC, and LAML (Figure 4A, 4B). The expression levels of immune checkpoint-related genes were negatively correlated with SUSD4 expression level in GBM, LGG, SARC, BRCA, LUSC, and BLCA.

Chemokines and their receptors recruit different subpopulations of immune cells into the TME, which are associated with tumor progression and immunotherapeutic outcomes [7]. We analyzed the correlation of chemokines with SUSD4 expression level and found a positive correlation of chemokines with SUSD4 expression level in COREAD, THYM, UVM, THCA, and LIHC. CXCL17 and CX3CL1 were positively correlated with SUSD4 expression level in most types of cancer (Figure 4C). Genes of the major histocompatibility complex (MHC) are essential for immune response to infections. We evaluated the correlation between the expression levels of MHC-related genes and SUSD4 expression level in tumor cells and found that the expression levels of MHC-related genes were negatively correlated with SUSD4 expression level in LGG, MESO, BLCA, LUSC, ESCA, SARC, HNSC, BRCA, and KIRC (Figure 4D). However, the expression levels of MHC-related genes were positively correlated with SUSD4 expression level in UVM, COADREAD, LIHC, OV, THCA, LAML, PRAD, and PCPG.

Finally, we examined the correlation between the expression level of an immunoreceptor gene and SUSD4 expression level and found similarities with previous results. In THCA, KIRP, HNSC, LAML, LIHC, COADREAD, OV, PCPG, and PRAD, the correlations were positive, while in ESCA, LUSC, BLCA, and BRCA, the correlations were mainly negative.

Through correlation analysis between SUSD4 expression and immune-related genes, we found a general positive correlation between SUSD4 and immune-related genes in cancers where high expression of SUSD4 is a poor prognostic factor. Conversely, a significant negative correlation trend was observed in cancers with a better prognosis associated with high SUSD4 expression. Based on these findings, we propose that SUSD4 may have a mechanism that affects immune regulation and thus influences tumor prognosis.

### TME and immune cell infiltration analysis

Malignant tumor tissues encompass not only tumor cells but also normal epithelial and stromal cells, immune cells, and vascular cells associated with the tumor. Stromal cells are closely linked to tumor growth, disease progression, and tumor resistance. We utilized the ESTIMATE algorithm, which includes stromal score, immune score, and ESTIMATE score, to assess the relationship between SUSD4 expression level and the TME (Supplementary Figure 1A). We observed a positive correlation between SUSD4 expression and three immune scores in LAML, UVM, COADREAD, and THCA. However, it is noteworthy that the immune score and ESTIMATE score in UVM did not exhibit statistical significance, consistent with the previously mentioned correlation results with immune-related genes. Conversely, in LGG, SARC, MESO, BLCA, UCEC, LUSC, and BRCA, a significant negative correlation was noted between SUSD4 expression and the three immune scores.

Furthermore, we conducted an analysis of the correlation between SUSD4 expression and immune-infiltrating cells using three different algorithms. Supplementary Figure 1B displays the correlation between SUSD4 expression, calculated using the EPIC algorithm, and immune-infiltrating cells. We observed a positive correlation between CD4 T cells and SUSD4 expression in multiple cancers. Conversely, cancer-associated fibroblasts showed a negative correlation with SUSD4 in THYM, LUSC, BLCA, GBM, and SARC. Interestingly, in these particular types of cancer, higher expression of SUSD4 was associated with better prognosis. However, no statistically significant correlation was found in CHOL, LUAD, UCEC, ACC, STAD, and KICH. Supplementary Figure 1C, 1D present the correlation analysis between SUSD4 expression and tumor immune-infiltrating cells using the TIMER algorithm and MCP counter algorithm, respectively. Similar results were also observed in THYM, LGG, SARC, LUSC, BLCA, and BRCA, where a negative correlation was observed with various immune cell infiltration scores. Conversely, significant positive correlations were found in COADREAD, LAML, PRAD, LIHC, and KIRP. These results suggest that SUSD4 may influence tumor immune infiltration, and there is a trend that cancers with high infiltration scores and high SUSD4 expression may have a poorer prognosis.

### Tumor heterogeneity analysis

**Figure 5:**
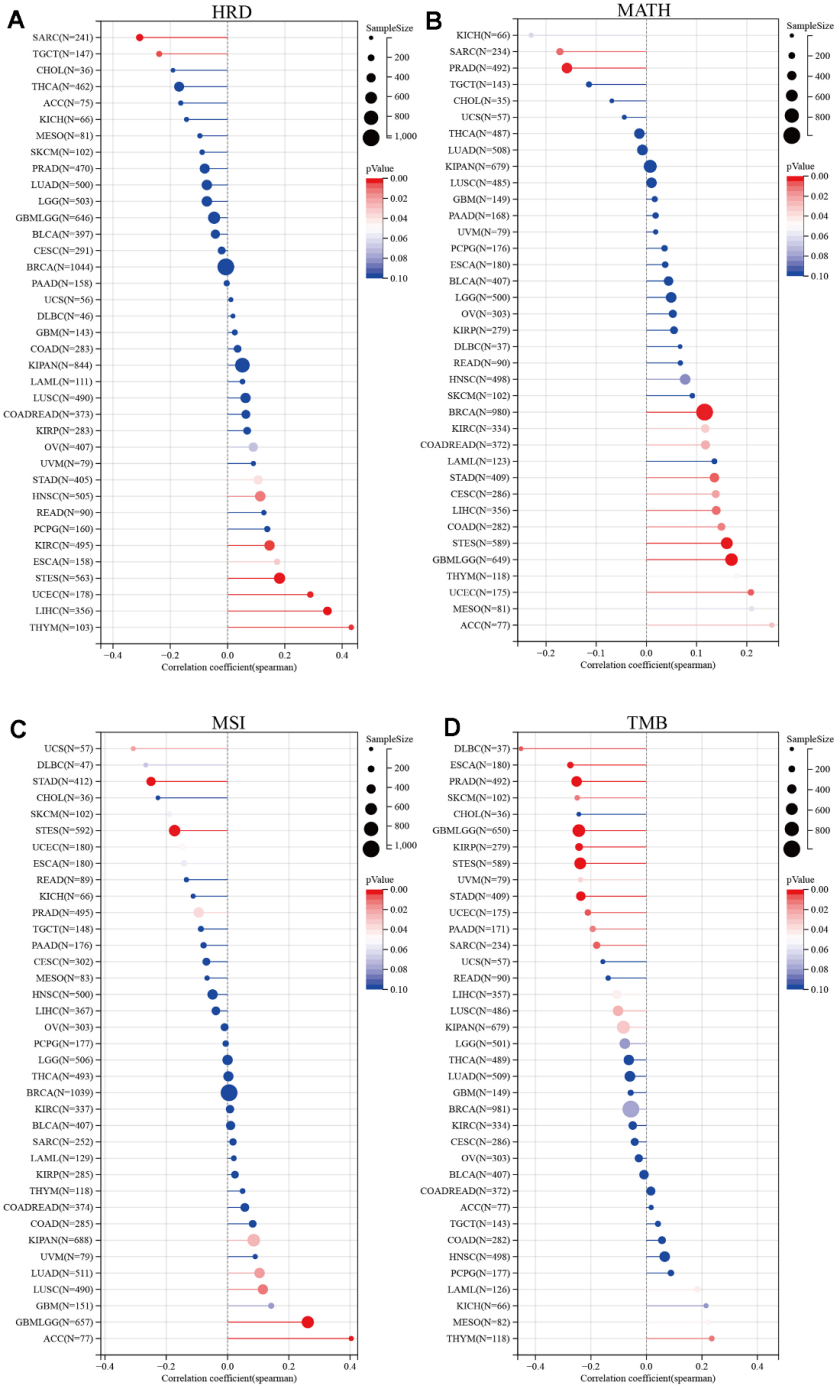
**Relationship between tumor heterogeneity and SUSD4 expression.** Lollipop plot illustrating the relationship between (**A**) Homologous recombination deficiency, (**B**) mutant-allele tumor heterogeneity, (**C**) Microsatellite Instability, and (**D**) Tumor mutational burden and SUSD4 expression. The color of dots is used to distinguish significance, and red represents P < 0.05. HRD, Homologous recombination deficiency. MATH, mutant-allele tumor heterogeneity. MSI, Microsatellite Instability. TMB, Tumor mutational burden. P-values were adjusted by Benjamini Hochberg (BH) method.

Cancer therapy has made significant advances, but the persistence of tumor heterogeneity poses a barrier to successful treatment. We evaluated the correlation between the expression level of SUSD4 and tumor heterogeneity using four metrics: microsatellite instability (MSI), tumor mutational burden (TMB), mutational burden-assessed tumor heterogeneity (MATH), and homologous recombination deficiency (HRD). Homologous recombination is a crucial process involved in DNA repair and replication. When DNA damage cannot be repaired through homologous recombination repair, it results in homologous recombination deficiency [8]. As displayed in Figure 5A, SUSD4 expression level was negatively correlated with HRD in SARC and TGCT, while positively correlated in STAD, HNSC, KIRC, ESCA, STES, UCEC, LIHC, and THYM. MATH is an algorithm used to calculate intratumor genetic heterogeneity, where higher values indicate a worse prognosis [9, 10]. The relationship between MATH and SUSD4 expression level was significantly correlated in 13 tumors, with a positive correlation in 11 tumors and a negative correlation in 2 tumors (Figure 5B). Microsatellite instability (MSI) arises from defects in the mismatch repair system, leading to hypermutation patterns. MSI is frequently utilized to guide treatment decisions, such as in colorectal cancer, where immune checkpoint blockade treatment decisions are made based on a patient’s MSI status [11]. Figure 5C, 5D display the relationship between SUSD4 expression level and MSI and TMB, respectively. A correlation was found between MSI and SUSD4 expression level in 10 tumors. However, there were negative and positive correlations between SUSD4 expression level and TMB in only 3 tumors.

### Genetic alteration analysis

**Figure 6:**
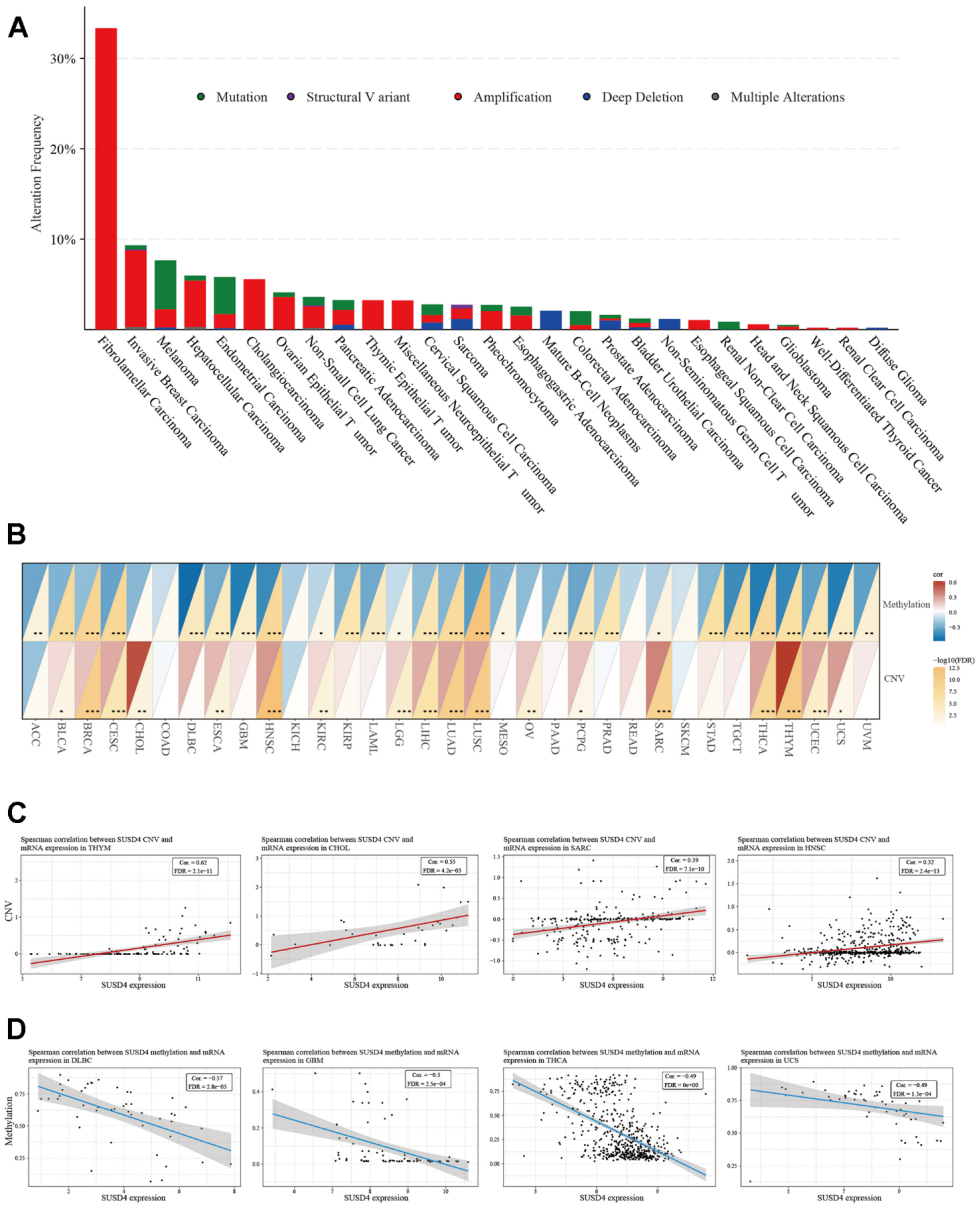
**Relationship between SUSD4 expression and gene alterations.** (**A**) The stacked bar plot shows the type and frequency of genetic changes in SUSD4. (**B**) Heatmap of correlation between methylation and copy number variation and SUSD4 expression. (**C**) Correlation between SUSD4 expression and CNV in THYM, CHOL, SARC and HNSC. (**D**) Correlation between SUSD4 expression and methylation in DLBC, GBM, THCA and USC. *p < 0.05, **p < 0.01, and ***p < 0.001. CNV, copy number variation. P-values were adjusted by false discovery rate (FDR) method.

Our analysis of SUSD4 genetic alterations in pan-cancer datasets from TCGA was conducted using the cBioPortal. The results revealed a relatively high overall frequency of SUSD4 alterations in various types of cancer, primarily driven by copy number variations (CNVs). Notably, fibrolamellar carcinoma exhibited the highest frequency, surpassing 30% (Figure 6A). Considering that CNAs may be associated with cancer progression, we investigated the association between SUSD4 expression level and CNV. Our analysis demonstrated a positive correlation between CNV and SUSD4 expression level across diverse types of cancer (Figure 6B). Furthermore, we assessed the relationship between SUSD4 expression level and DNA methylation, as DNA methylation is known to play a role in the epigenetic mechanisms of cancer. Interestingly, we found a negative correlation between SUSD4 expression level and DNA methylation mutations in 18 types of cancer (Figure 6C). Additionally, our analysis revealed strong correlations between SUSD4 expression level and both CNV and DNA methylation in various cancers (Figure 6D, 6E).

### Drug sensitivity analysis

**Figure 7:**
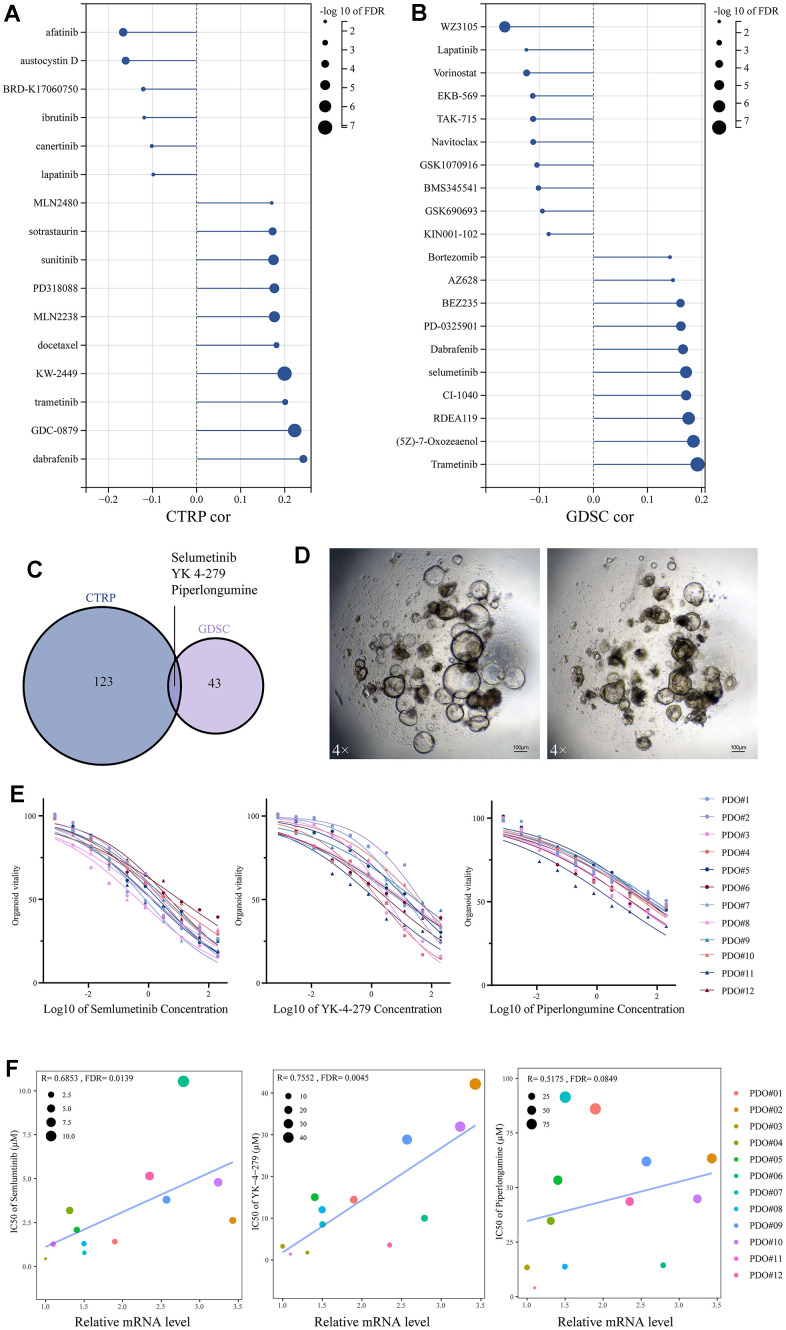
**Drug sensitivity analysis based on SUSD4 expression.** (**A**) CTRP database and (**B**) GDSC database. (**C**) Venn diagram between two drug datasets. (**D**) Colorectal cancer organoids under a 4X microscope, the length of the bar is 100μM. (**E**) IC50 for three drugs in 12 organoids. (**F**) Spearman correlation between mRNA level of SUSD4 and IC50 of three drugs, the size and color of the dots represent IC50 and organoids’ ID respectively. CTRP, cancer therapeutics response portal. GSDC, genomics of drug sensitivity in cancer. IC50, half maximal inhibitory concentration. P-values were adjusted by false discovery rate (FDR) method.

Through tumor heterogeneity analysis, it was hypothesized that SUSD4 has the potential to be an immunotherapeutic target. We used the GDSC database to analyze potential drugs. Figure 7A, 7B demonstrate that the drugs in CTRP and GDSC databases are strongly correlated with SUSD4 expression level. Three drugs were correlated with SUSD4 expression in both drug databases through correlation analysis, including Selumetinib, YK-4-279, and piperlongumine (Figure 7C). Selumetinib is a selective, non-ATP-competitive MEK1/2 inhibitor used for the treatment of neurofibromatosis. YK-4-279 functions by blocking the interaction of the oncogenic protein EWS-FLI1 and is thought to have anti-tumor effects and induce apoptosis [12]. Piperlongumine, a derivative from Piper longum, is reported to have antitumor activities [13]. To verify the correlation between SUSD4 expression and the three drugs, colorectal cancer samples were harvested, and organoid cultures were performed. Figure 7D illustrates the colorectal cancer organoids, with the left panel showing normal growth and the right panel indicating changes observed one day after delivering Selumetinib. We conducted IC50 characterization of the three drugs in 12 organoids, revealing substantial heterogeneity in drug sensitivity among different patient-derived organoids. For Selumetinib, the lowest IC50 was observed in PDO#3 at 0.44 μM, while the highest IC50 was recorded in PDO#6 at 10.54 μM. Among the 12 organoids, relative insensitivity to YK-4-279 was noted, with the lowest IC50 being 1.42 μM for PDO#11 and the highest IC50 recorded at 42.16 μM for PDO#02. There was a trend towards resistance to piperlongumine in colorectal organoids, with the lowest IC50 measured at 4.04 μM for PDO#11 and the most resistant being PDO#07 with an IC50 of 91.45 μM.

To verify the relationship between SUSD4 expression and the sensitivity of the above drugs, a correlation analysis was performed based on the SUSD4 expression levels of the samples and IC50 values. As depicted in Figure 7F, the IC50 of both Selumetinib and YK-4-279 tended to increase statistically as the expression of SUSD4 increased. However, in the correlation analysis of piperlongumine with SUSD4 expression, the FDR value was not statistically significant, although the R value reached 0.5175. Additionally, we found that all three drugs exhibited lower IC50 values and higher sensitivity to drug treatment in organoids with the lowest relative expression of PDO#02 and PDO#12.

### Enrichment and pathway analyses

**Figure 8:**
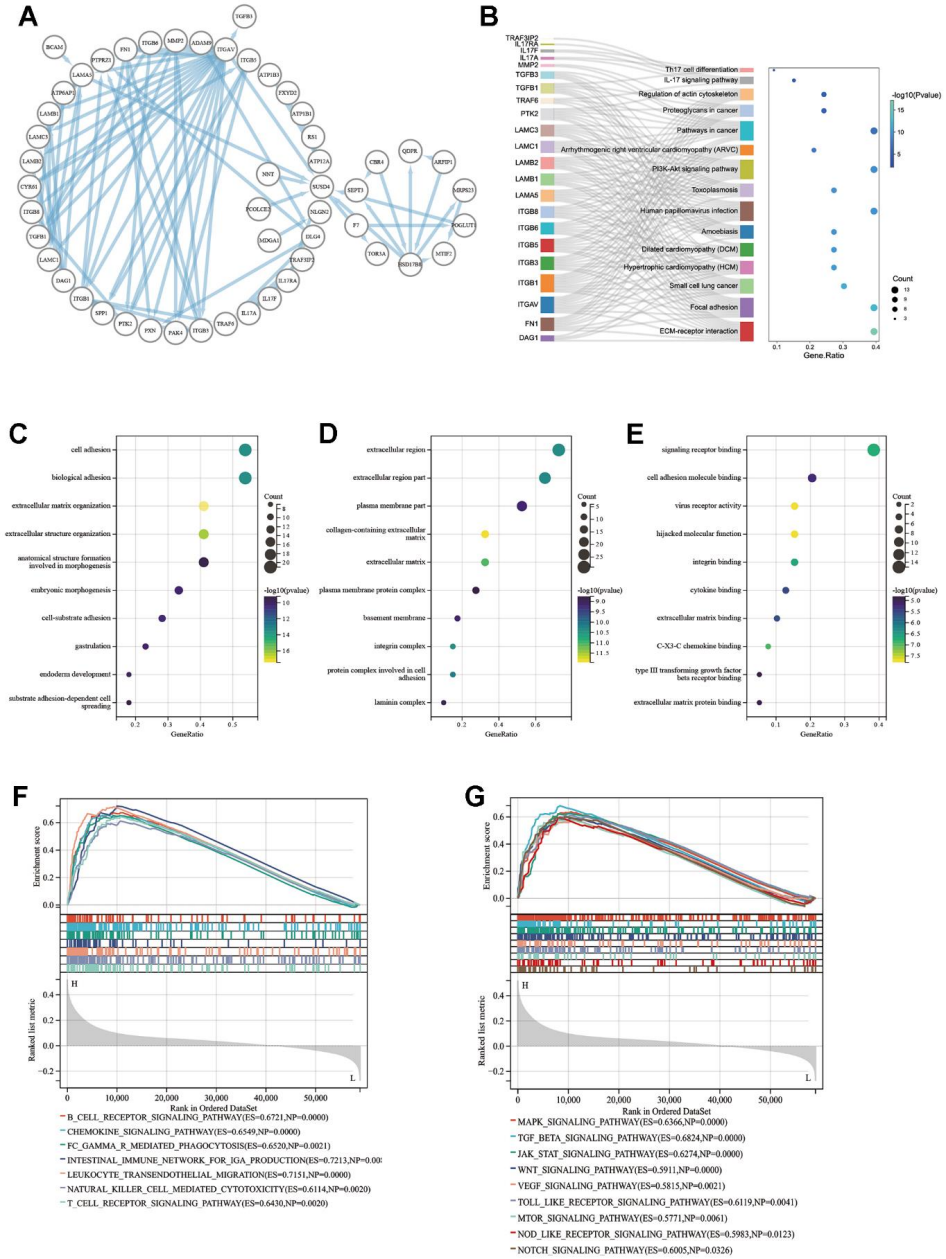
**Enrichment analysis result of SUSD4.** (**A**) Protein-protein interaction analysis of SUSD4 replotted by Cytoscape. (**B**) Sankey plot of KEGG enrichment analysis result. Biological process (**C**), cellular competent (**D**) and molecular function (**E**) result of GO enrichment analysis. Immune-related pathways (**F**) and cancer pathways (**G**) were enriched by GSEA in colorectal cancer. KEGG, Kyoto encyclopedia of genes and genomes. GO, gene ontology. GSEA, gene set enrichment analysis. P-values of KEGG and GO were adjusted by Benjamini Hochberg (BH) method.

Results showed that the expression level of SUSD4 was associated with prognosis, immunity, and heterogeneity in some types of cancer, and we attempted to utilize enrichment analysis to predict the possible mechanism of SUSD4. First, we constructed the PPI network using the STRING database and visualized it using Cytoscape software (Figure 8A). Figure 8B displays the results of the KEGG pathway analysis, revealing that the main functions of the proteins interacting with SUSD4 were related to cancer and immune-related pathways. Figure 9C–9E illustrate the results of the GO enrichment analysis, including biological processes, cellular components, and molecular functions, which are similar to the outcomes of the KEGG pathway analysis.

**Figure 9:**
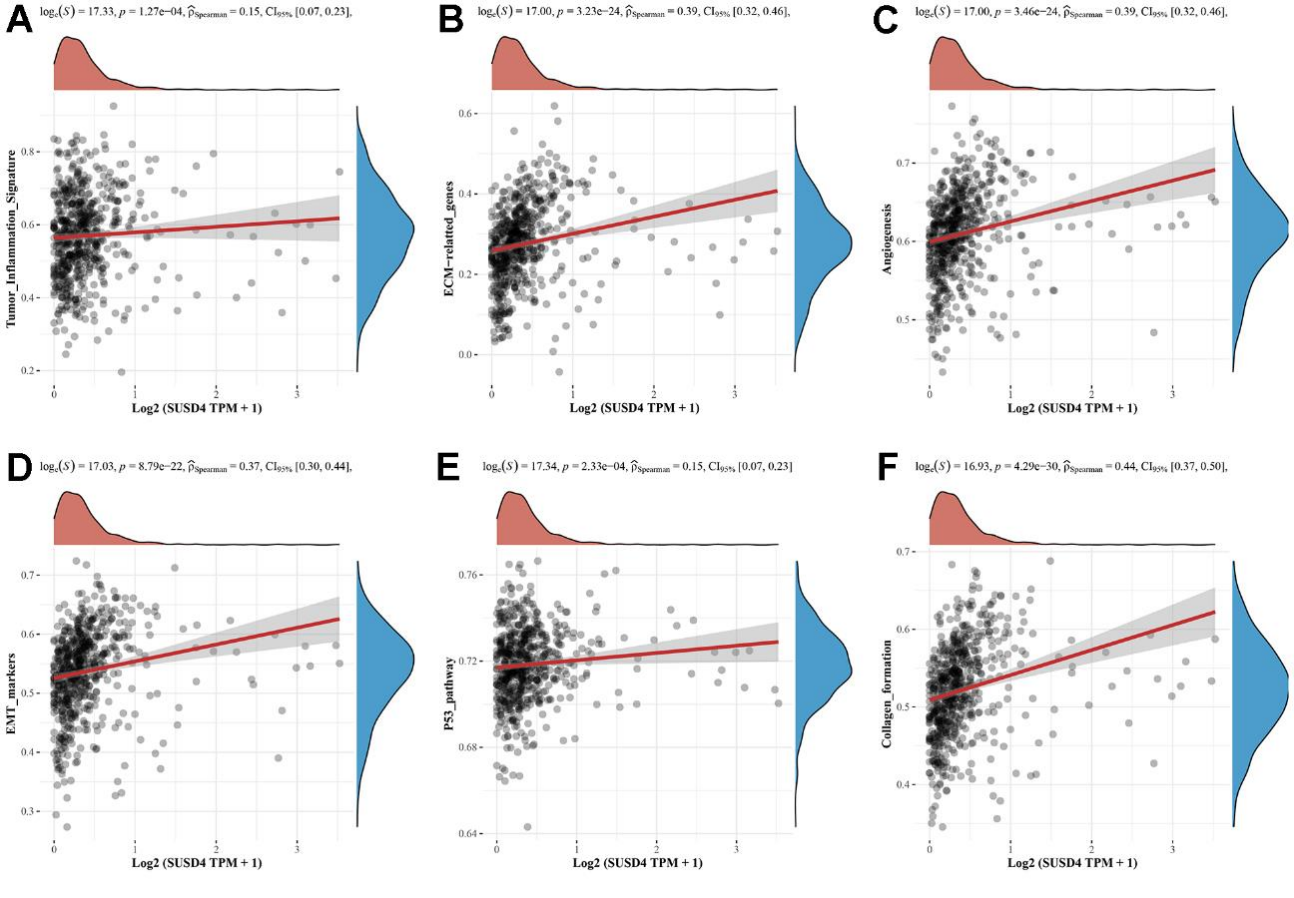
**Correlation analysis between SUSD4 expression and pathway signatures in COADREAD.** (**A**) Tumor inflammation signature. (**B**) ECM−related genes signature. (**C**) Angiogenesis signature. (**D**) EMT markers signature. (**E**) P53 pathway signature. (**F**) Collagen formation signature.

**Figure 9:**
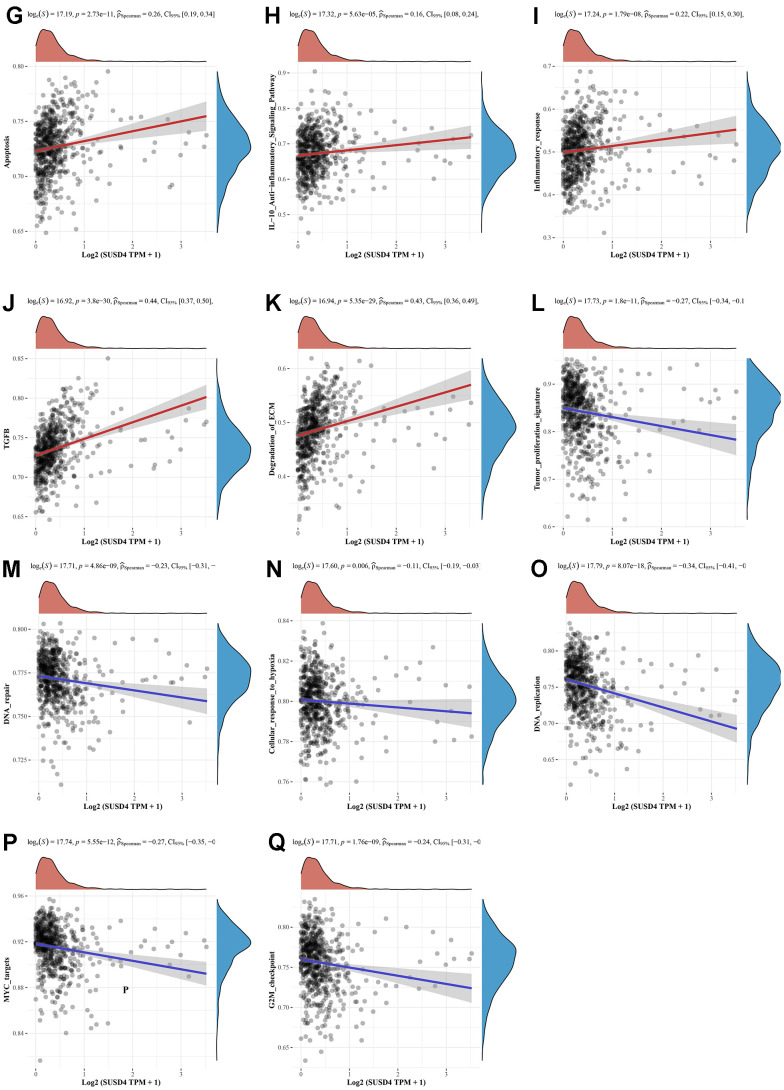
**Correlation analysis between SUSD4 expression and pathway signatures in COADREAD.** (**G**) Apoptosis signature. (**H**) IL−10 Anti−inflammatory Signaling Pathway signature. (**I**) Inflammatory response signature. (**J**) TGFB signature. (**K**) Degradation of ECM signature. (**L**) Tumor proliferation signature. (**M**) DNA repair signature. (**N**) Cellular response to hypoxia signature. (**O**) DNA replication signature. (**P**) MYC targets signature. (**Q**) G2M checkpoint signature. ECM, extracellular matrix. EMT, Epithelial-Mesenchymal Transition. IL-10, interleukin-10. TGFB, transforming growth factor-β.

COREAD was prominent in previous results, both in terms of immune correlation, survival prognosis, and tumor heterogeneity in association with SUSD4 expression level. We performed GSEA using COADREAD RNA expression matrix and clinical information from TCGA. Figure 8F, 8G show the enriched immune-related pathways and cancer-related pathways, respectively.

We hypothesized that SUSD4 expression level could be closely associated with colorectal carcinogenesis progression, and the enrichment scores of each sample on common cancer-related pathways were calculated sequentially according to the ssGSEA algorithm to obtain the association between samples and pathways. The results discovered that SUSD4 expression level was positively correlated with tumor inflammation, extracellular matrix (ECM), angiogenesis, epithelial– mesenchymal transition (EMT), p53 pathway, collagen formation, apoptosis, IL-10, inflammatory response, TGF-β pathway, and degradation of ECM (Figure 9A–9K). In addition, SUSD4 expression level was negatively associated with cancer proliferation, DNA repair, cellular response to hypoxia, DNA replication, MYC target genes, and G2M DNA damage checkpoint (Figure 9L–9Q).

### Exploring the impact of SUSD4 knockdown *in vivo* and *in vitro*

**Figure 10:**
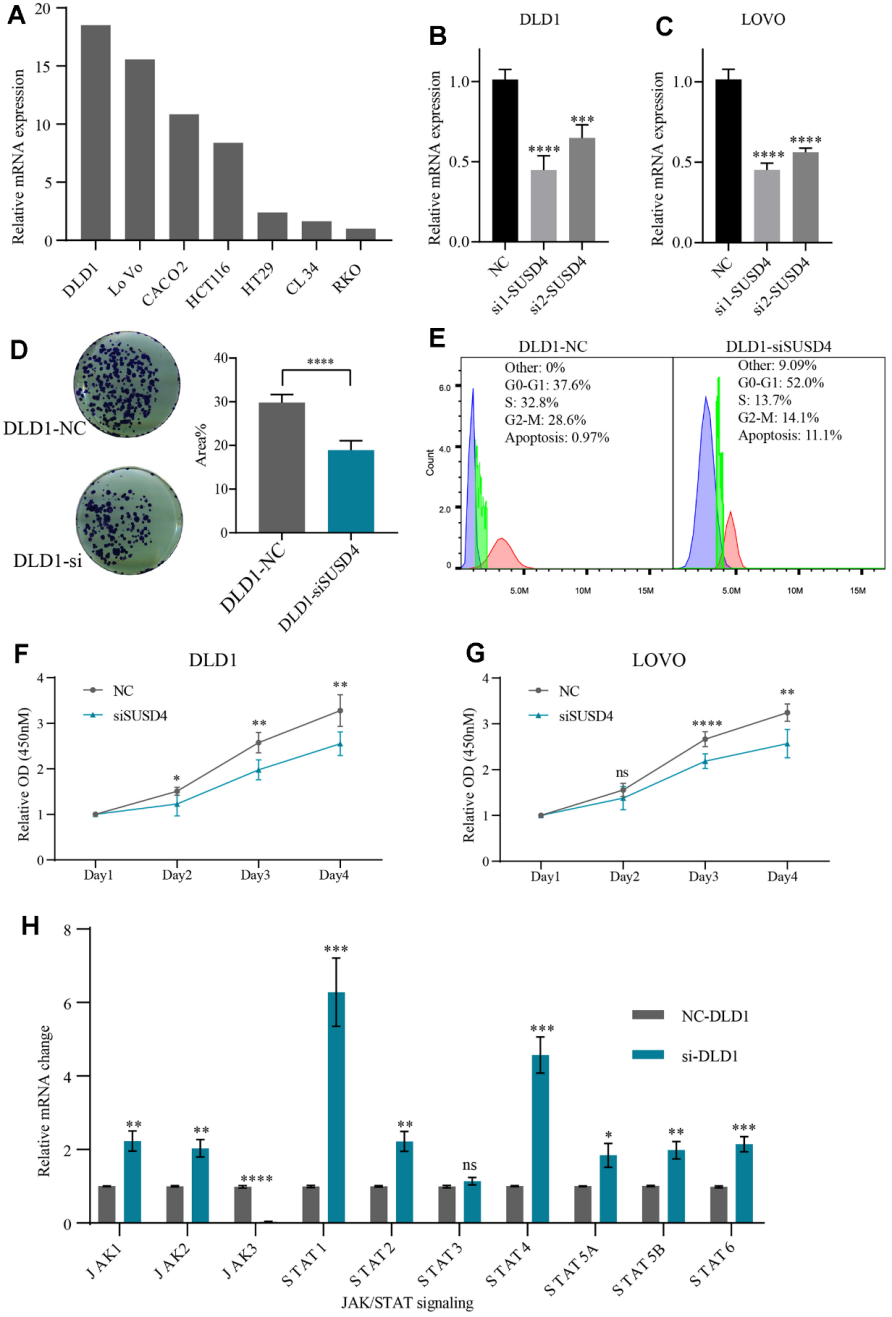
**Knockdown of SUSD4 inhibits the cell proliferation and impacts on JAK/STAT pathway.** (**A**) Relative mRNA level of SUSD4 in multiple colorectal cancer cell lines. (**B**) Validation of siRNA knockdown efficiency in DLD1. (**C**) Validation of siRNA knockdown efficiency in LOVO. (**D**) Clonogenic assay and comparison of colony areas. (**E**) Cell cycle assay. (**F**) Cell proliferation assays in DLD1. (**G**) Cell proliferation assays in LOVO. (**H**) Relative mRNA changes of JAK/STAT pathway key gene between the negative control and knockdown group in DLD1. *p<0.05, **p<0.01, ***p<0.001, ****p<0.0001.

Given that patients with high SUSD4 expression in CRC exhibit a poorer prognosis, despite its expression levels not being higher in CRC tissues, we proceeded to knock down and interfere with SUSD4 using CRC cell lines. Firstly, we verified the expression of SUSD4 in various colorectal cancer cell lines and found the highest expression in DLD1 and LOVO (Figure 10A). Therefore, we performed knockdown validation using DLD1 and LOVO. As shown in Figure 10B, 10C, the knockdown efficiency of si1 was higher in both cell lines, thus si1 was used for the next experiment.

By clone formation assay, we found that the colony area was lower after knockdown of SUSD4 (Figure 10D). CCK8 further corroborates the results of clone formation, with significant differences occurring on days three and four after knockdown of the target gene (Figure 10F, 10G). Further, we conducted subcutaneous tumor implantation experiments in nude mice. Supplementary Figure 2A illustrates the expression levels in DLD1 cells post-shRNA knockdown, and Supplementary Figure 2B presents images comparing tumors from sh-SUSD4 and sh-NC groups. When evaluating tumor volume and weight, mice with sh-SUSD4-derived subcutaneous tumors exhibited smaller tumor volumes and lighter weights compared to the control group (Supplementary Figure 2C, 2D). Given the effect of knockdown of SUSD4 on cell proliferation, we speculate that cell cycle changes also occur when SUSD4 is knocked down. Figure 10E reveals the cell cycle changes following knockdown of SUSD4, with an increased proportion of cells in G0-G1 phase and apoptosis and a decreased proportion of cells in G2-M phase compared to the NC group.

Building upon the findings from the previous section, our investigation suggests a potential association between SUSD4 and the JAK/STAT signaling pathway. Thus, we verified the mRNA changes in key genes of the JAK/STAT pathway after knockdown of SUSD4 compared to the NC group. We found no obvious changes in mRNA levels except for STAT3. JAK3 was dramatically reduced after knockdown of SUSD4, while other critical genes were significantly elevated (Figure 10H). To further confirm the impact of SUSD4 knockdown on JAK3, we conducted Western Blot validation. As shown in Supplementary Figure 2E, following SUSD4 knockdown, both SUSD4 and JAK3 protein levels significantly decreased. Given that the primary focus of this study does not involve elucidating the specific mechanisms of SUSD4 in cancer, further exploration in this regard was not pursued.

## DISCUSSION

The complement system constitutes a meticulously orchestrated network of proteins indispensable for host defense and inflammatory processes [14, 15]. Its functionality extends beyond the activation of macrophages, neutrophils, eosinophils, and basophils [16, 17], encompassing a role in modulating the adaptive immune response and potentially shaping T cell reactions to tumors [3, 18, 19]. In this intricate milieu, Sushi domain-containing protein 4 emerges as a pivotal complement regulator primarily tasked with modulating complement system activity. Building upon this premise, we postulate that SUSD4 may exert a discernible influence on tumorigenesis and cancer progression.

To date, scant attention has been devoted to the exploration of Sushi domain-containing protein 4 (SUSD4), with a paucity of studies investigating its implications across diverse cancer types. Existing research has predominantly centered on assessing SUSD4 expression levels in lung adenocarcinoma and breast cancer contexts [5, 20]. Thus, recognizing this void in the literature, our study endeavors to bridge this gap by methodically scrutinizing the correlation between SUSD4 expression levels and a comprehensive spectrum of cancer types. Through this systematic approach, we aim to unveil novel perspectives on the potential relevance of SUSD4 in the landscape of cancer research.

Our systematic pan-cancer analysis has elucidated a dualistic nature in the role of Sushi domain-containing protein 4 (SUSD4) expression within tumors. Upon comparing SUSD4 expression levels between cancerous and normal tissues, we observed pronounced elevation exclusively in select cancer types. Moreover, our survival analysis unveiled two discernible impacts of SUSD4 expression levels on prognosis, thereby underscoring its potential utility as a prognostic biomarker across diverse cancer contexts.

To explore the dual effects of SUSD4 expression on tumors, we assessed the correlation between various immune-related genes and SUSD4 expression. Our findings revealed a general negative correlation, suggesting SUSD4 may serve as a protective prognostic factor. Englund et al. identified SUSD4 as a prognostic factor for breast cancer (BRCA), demonstrating that knocking down SUSD4 in breast tumor cell lines inhibits cell migration and invasion. However, our results did not indicate a significant effect of SUSD4 expression on BRCA prognosis. Nevertheless, analysis of the correlation between SUSD4 expression and immune cells showed a significant negative correlation in BRCA. Conversely, in colorectal adenocarcinoma, liver hepatocellular carcinoma, and kidney renal papillary cell carcinoma, high SUSD4 expression was significantly associated with poor prognosis and increased immune cell infiltration.

The correlation analysis results revealed a noteworthy pattern: SUSD4 expression exhibited positive correlations with most immune-related genes in COADREAD, UVM, DLBC, LAML, LIHC, PCPG, and PRAD, while negative correlations were observed in LGG, BRCA, BLCA, MESO, and LUSC. Overall, a prevailing trend emerged wherein the correlation between SUSD4 expression and immune-related genes tended to be positive across various cancer types. Additionally, our analysis of SUSD4 correlation with immune-related genes and tumor infiltration unveiled a consistent trend: in instances where elevated SUSD4 expression was linked to poorer prognosis, a positive correlation with immune-related genes and infiltrating cells was evident. Conversely, when heightened SUSD4 expression conferred a protective prognosis, a negative correlation was observed. These observations suggest a potential role for SUSD4 in tumor immunity, with consequential implications for patient prognosis. Accordingly, these findings underscore the need for further comprehensive investigation and discussion to elucidate the mechanistic underpinnings of SUSD4 in tumor immunity and its impact on patient outcomes.

To delve deeper into the multifaceted functions of SUSD4, we embarked on our investigation by constructing a protein-protein interaction (PPI) network. Subsequently, we conducted Gene Ontology (GO) and Kyoto Encyclopedia of Genes and Genomes (KEGG) pathway enrichment analyses based on the PPI network. Remarkably, our analyses revealed significant enrichment of numerous pathways related to both tumorigenesis and immune response, consistent with previous findings. This underscores the involvement of SUSD4 in these crucial biological processes.

Moreover, our exploration uncovered a distinctive association between SUSD4 and colorectal adenocarcinoma, prompting us to delve deeper into this connection from multiple perspectives. Consequently, we performed gene set enrichment analysis (GSEA) in COADREAD to unravel the potential mechanisms underlying the oncogenic actions of SUSD4 in colorectal cancer. Additionally, leveraging the single-sample gene set enrichment analysis (ssGSEA) algorithm, we corroborated the correlation between SUSD4 expression levels and prevalent cancer-related pathways in colorectal cancer. These findings provide further support to prior research implicating SUSD4 in immune responses and underscore its intricate interaction with multiple oncogenic pathways. Significantly, the regulatory correlations associated with SUSD4 were found to be dependent on the specific cancer type. These variations encompassed various regulatory levels, including genetic alterations, expression patterns, DNA methylation, immune-related genes, tumor microenvironment (TME), and pathway correlations. These disparities may have implications for differences in drug efficacy, treatment response, and survival rates among different cancer types.

To investigate the potential oncogenic role of SUSD4, we conducted knockdown experiments in colorectal cancer cell lines. The results indicated that diminishing SUSD4 expression led to reduced proliferation, suggesting that SUSD4 plays a regulatory role in cell proliferation *in vitro*. Corresponding outcomes were observed *in vivo*, where mice with SUSD4-disrupted expression developed smaller and lighter subcutaneous tumors. Considering the critical importance of the JAK/STAT signaling pathway in cytokine transduction and its close association with cell proliferation, migration, and apoptosis, we explored its potential involvement in SUSD4-mediated actions. Enrichment analysis suggested that SUSD4 might participate in the activation of the JAK/STAT pathway. These findings were corroborated through RT-qPCR experiments and Western Blot analysis, confirming a significant reduction in JAK3 expression following SUSD4 knockdown. Additionally, inhibiting JAK3/STAT3 in colorectal cancer cells promoted apoptosis and inhibited cell proliferation, aligning with previous results. These insights offer a more comprehensive understanding of SUSD4’s function in cancer biology, specifically in colorectal cancer, and highlight its interaction with the JAK/STAT signaling pathway.

Further investigation into these mechanisms holds promise for enhancing our understanding of the role of SUSD4 in cancer progression, as well as for guiding the development of targeted therapeutic strategies [21]. Interestingly, our results revealed a significant increase in the mRNA level of STAT1 following SUSD4 knockdown. Notably, high expression of STAT1 in colorectal cancer has been associated with a favorable prognosis [22]. These findings suggest a potential link between SUSD4, STAT1, and the clinical outcomes of colorectal cancer.

Additional research is imperative to unveil the underlying mechanisms and delve into the therapeutic implications of this association. Comprehending the intricate interplay among SUSD4, JAK/STAT signaling, and colorectal cancer prognosis offers potential for identifying novel therapeutic targets and devising personalized treatment strategies for patients.

Patient-derived organoids, which better represent tumor heterogeneity compared to established tumor cell lines, are widely used in drug screening. Through organoid drug analysis, we confirmed the correlation between SUSD4 expression and the response to Selumetinib and YK-4-279. These findings support the potential of SUSD4 as a therapeutic marker for colorectal cancer.

However, it is crucial to acknowledge the limitations of this study. The depth of gene function validation and the effects on pathways may not have been fully explored. Nonetheless, the primary aim of this study was to deliver a comprehensive pan-cancer analysis of SUSD4, and more intricate validation of gene function will be pursued in future investigations. Continued exploration of the functional roles and molecular mechanisms linked with SUSD4 is imperative to attain a profound comprehension of its relevance in cancer biology. This endeavor will facilitate the development of targeted therapies and personalized treatment strategies for patients afflicted with colorectal cancer and potentially other malignancies as well.

In conclusion, our research explores the role of Sushi domain-containing protein 4 across various cancer types, particularly focusing on its involvement in tumor immunity and cancer progression. Through systematic pan-cancer analysis, our study reveals a dualistic nature of SUSD4 expression in tumors, impacting prognosis differently depending on the cancer type. Correlation analyses with immune-related genes highlight SUSD4’s potential as a prognostic biomarker and its association with tumor immunity. Protein-protein interaction network and enrichment analyses indicate SUSD4’s involvement in tumorigenesis and immune response pathways. In-depth investigation in colorectal cancer uncovers SUSD4’s role in cell proliferation via the JAK/STAT signaling pathway and its potential as a therapeutic marker. The study underscores the significance of SUSD4 in cancer biology, suggesting it as a promising therapeutic target and prognostic indicator across diverse cancer types, while acknowledging the need for further mechanistic exploration and validation.

## MATERIALS AND METHODS

### Data collection and processing

A standardized pan-cancer dataset was obtained from the UCSC Xena functional genomics explorer (https://xenabrowser.net/), followed by extraction of the expression data of ENSG00000143502 (*SUSD4*) gene. The GTEx (Genotype-Tissue Expression) data and TCGA (The Cancer Genome Atlas) data available in the XENA database have undergone batch correction, enabling direct comparisons between the two datasets. For each expression value, log2(x+1) transformation was performed.

### Gene expression and survival analysis

The cancer cell line expression matrix was acquired from the Cancer Cell Line Encyclopedia (CCLE) database (https://portals.broadinstitute.org/ccle/about), and analyzed by “ggplot2” R package (ver. 3.3.3).

Calculating the differential expression between normal and tumor samples was done using unpaired Wilcoxon tests using the R software. Additionally, we obtained a high-quality prognostic dataset from The Cancer Genome Atlas (TCGA), as previously reported by Liu et al. [23]. The Cox proportional-hazards regression model was subsequently established using the coxph module of the “survival” R package (ver. 3.2-7) to analyze the inner link between gene expression and prognosis. We also calculated the optimal cut-off value of risk score using the “maxstat” R package (ver. 0.7-25), in which the minimum sample size was set to greater than 25% and the maximum sample size was set to lower than 75%.

### Analysis of immune-related genes

We extracted expression data of 150 marker genes of the 5 classes of immune pathways (chemokine, receptor, MHC, immunoinhibitor, immunostimulator) from the downloaded TCGA dataset, and then, analyzed the Spearman’s correlation of SUSD4 expression with these genes. Immune checkpoints consisting of inhibitory and stimulatory pathways were obtained from the Immune Landscape of Cancer [24], and the Spearman’s correlation between these two classes of genes and the SUSD4 expression was analyzed using TCGA database.

### TME and infiltration of immune cells

The “ESTIMATE” R package (ver. 1.0.13) calculates stromal, immune, and ESTIMATE scores for each patient based on gene expression [25]. We re-evaluated the scores of each patient’s immune cells using the Timer method via the “IOBR” R package (ver. 0.99.9) [26, 27]. For the immune cell infiltration score, we employed three algorithms, namely TIMER [26], EPIC [28], and MCP counter [29], and utilized IOBR for calculation and analysis.

### Tumor heterogeneity analysis

MuTect2 software processed the level 4 simple nucleotide variation dataset downloaded from TCGA. Besides, the tumor mutational burden (TMB) and mutant-allele tumor heterogeneity (MATH) for each tumor were calculated using the TMB and inferHeterogeneity functions of the “maftools” R package (ver. 2.8.05), and the TMB and MATH scores were combined with gene expression data [30]. Homologous recombination deficiency (HRD) and microsatellite instability (MSI) scores for each tumor were obtained from previous studies [24, 31]. Then, the correlation of HRD and MSI scores with gene expression data was assessed using Spearman’s correlation analysis.

### Genetic alteration analysis

We analyzed genetic alterations using the cBio Cancer Genomics Portal (http://cbioportal.org), an open-access resource for interactive exploration of multidimensional cancer genomic datasets [32]. In addition, “Cancer Types Summary” sub-menu was used to analyze and visualize genetic alteration frequencies. To assess the relationship between DNA methylation and copy number alteration (CNA) profile of SUSD4, the “mutation” module in the Gene Set Cancer Analyses (GSCA) (http://bioinfo.life.hust.edu.cn/GSCA/#/mutation) was utilized [33].

### Drug sensitivity analysis *in silico*

Drug sensitivity data of various tumor cell lines and mRNA expression levels were obtained from the Genomics of Drug Sensitivity in Cancer (GDSC) (www.cancerrxgene.org/) and Cancer Therapeutics Response Portal (CTRP)(https://portals.broadinstitute.org/ctrp/) [34–39]. Pearson’s test was performed to discovery the correlation between gene expression and half-maximal inhibitory concentration (IC50). False discovery rate (FDR)-adjusted p-values were calculated. RNA-sequencing expression (level three) profiles and the parallel clinical records were downloaded from TCGA, and “pRRophetic” R package was applied to calculate the chemotherapy sensitivity of each sample. Based on ridge regression, the IC50 was calculated. All parameters were set as the default values. We removed the batch effect using “ComBat” and set the tissue type to “allSoldTumours” We summarized duplicate gene expression by calculating the mean.

### Gene ontology (GO) and Kyoto Encyclopedia of Genes and Genomes (KEGG) pathway enrichment analysis

In order to construct the protein-protein interaction network (PPI), we used the STRING database (https://cn.string-db.org/) and plotted the results with Cytoscape (ver. 3.9.0) [40–43]. As for STRING parameters, the minimum interaction score was 0.15, and the top 50 relative proteins were obtained. The “clusterProfiler” R package was used to perform GO and KEGG pathway enrichment analyses, and the results were illustrated using the “Riverplot” R package for visual presentation of Sankey.

For gene set enrichment analysis (GSEA), the GSEA software (ver. 3.0) was utilized, and samples were grouped by SUSD4 expression level (cut off value is 50%) [44]. KEGG symbol matrix was acquired from the Molecular Signatures Database (http://www.gsea-msigdb.org/gsea/downloads.jsp) to evaluate the potential pathways and mechanisms based on gene expression profiles and groupings, in which the minimum and maximum gene set function was set to 10 and 500 re-samplings, respectively [45].

### Pathway correlation analysis

The pathway correlation analysis was performed by “GSVA” R package, the ssGSEA method was employed, and the correlation between gene and pathway scores was finally analyzed by the Spearman’s correlation analysis [46]. The AmiGO database (http://amigo.geneontology.org/amigo/) was used to analyze the GO terms for selected genes to verify the accuracy and annotate biofunctions of identified genes. More specifically, the website https://www.aclbi.com/ is used for conducting correlation analysis between pathway scores and gene expression levels.

### Cell culture

All the cell lines (DLD1, LOVO) used in this study were purchased from the American Type Culture Collection (ATCC). Cells were grown in a 37° C incubator using DMEM medium (Gibco, USA, Cat.11965092) containing 10% fetal bovine serum (Gibco, USA, Cat. 10091148) and 5% CO_2_.

### Organoid culture

Patients with tumors have a markedly improved overall survival rate after chemotherapy. It is important to note, however, that cancer cell chemosensitivity is highly heterogeneous. Organoid cultures are now thought to predict sensitivity to radiotherapy in patients with CRC [47]. A total of 12 colorectal cancer organoids were harvested. Briefly, after obtaining the cancer tissue, the tissue is first thoroughly washed using a washing buffer. The tissue is then cut up and added to the tissue digestion solution. The tumour cells were filtered using a 70 μM filter, resuspended again using the washing buffer and centrifuged. After removal of the supernatant, the Matrigel (BD, Cat.356234) was added for resuspension. Finally, the cell suspension was inoculated into 48-well plates (Corning 3300). Organoid culture medium purchased from STEMCELL (IntestiCult™ Organoid Growth Medium (Human), Cat.06010).

### Cell transfection

We have cloned a full-length plasmid DNA from the SUSD4 deletion construct obtained from GENECHEM Company (https://www.genechem.com.cn/). Vazyme Lipofectamine (ExFect® Transfection Reagent) was used for plasmid transfection and siRNA interference for gene knockdown. The SUSD4 siRNA sequences were as follows: (1) AUUUGAUGCAGUAGACUUGAU, CAAGUCUACUGCAUCAAAUCA, and (2) AAUGUAUGGUUCAAUUUGGGG, CCAAAUUGAACCAUACAUUUA. The SUSD4 shRNA sequences were as follows: (1) CGATGATGGAACGTGGAATAA, (2) ATGGTGAGTCACGGAGATTTC. For stable shRNA-transfected SUSD4 knockdown cell lines, we utilized puromycin for screen.

### Proliferation assay

According to the manufacturer’s instructions (CCK-8 Cell Counting Kit, Vazyme, China), cell counting kit 8 (CCK8) was also used to evaluate cell proliferation. In the following step, samples were analyzed for absorbance using a Spark 20M microplate reader (Tecan Instruments) at 450 nm. The plate clonogenic assay was used to determine colony forming ability. And the experimental procedure was referred to previous studies [48]. After the cell colonies were formed, photographs were taken using a scanner (Microtek, China) and then the colony areas were calculated using ImageJ (https://imagej.nih.gov/ij/).

### Drug sensitivity assay *in vitro*

Selumetinib (HY-50706), YK-4-279 (HY-14507), and piperlongumine (HY-N2329) were purchased from MCE (https://www.medchemexpress.cn/). DMSO is used as a solvent and the maximum concentration of DMSO during cell culture does not exceed 0.5%. Organoid viability assay using the CellTiter-Glo® 3D Cell Viability Assay (Promega, G9681). All drug sensitivity verifications were carried out on the third day after drug was delivered.

### Cell cycle assay

Cell Cycle and Apoptosis Analysis Kit (C1052) purchased from https://www.beyotime.com/ to detect cell cycle and apoptosis. The assay was performed according to the manufacturer’s instructions. First, after performing transfection, cells and supernatant were collected, then cells were fixed using pre-cooled 70% ethanol and then stained with propidium iodide. The cells were resuspended and then assayed using a Beckman Coulter (CytoFLEX LX). Analyses were carried out with Flow Jo Software (FLOWJO, LLC).

### *In vivo* model

To further validate the function of SUSD4, we employed a CDX model for *in vivo* validation. Subcutaneous tumor experiments were conducted using 4-5-week-old female nude mice. Firstly, cells were prepared by culturing them until confluency reached around 80%. Then, on the day prior to tumor inoculation, fresh culture medium was replenished. After washing the cells with PBS, cell counting was performed. To avoid immunogenic reactions, cells were resuspended in 100μL PBS, and each mouse was injected with 5×10^6^ cells. Tumor volume was calculated using the formula: length × (0.5 × width)^2^.

### RNA isolation and quantitative real-time PCR (qRT–PCR)

FastPure Cell/Tissue Total RNA Isolation Kit V2 (RC112) from Vazyme® used to extract RNA from cell. HiScript® II Q RT SuperMix for qPCR (+gDNA wiper) (R223) from Vazyme® used to reverse transcription. ChamQ Universal SYBR qPCR Master Mix (Q711) from Vazyme® used to qPCR validation. Primer sequences is available in Supplementary Material (Supplementary Table 2).

### Western blot and protein extraction

To perform Western blotting (WB) on cultured cells, cells are first grown in appropriate culture dishes until reaching the desired confluency. Following this, the cells are rinsed with phosphate-buffered saline and detached from the culture dish using trypsin-EDTA. The detached cells are then collected by centrifugation, and the resulting cell pellet is resuspended in ice-cold lysis buffer (Beyotime, Cat.P0013B) containing protease inhibitors (Beyotime, Cat.ST506). After incubating the cell lysate on ice for 15-30 minutes with intermittent vortexing to ensure complete lysis, the lysate is centrifuged (12000g for 15 minutes at 4° C) to remove cell debris, and the supernatant containing the protein lysate is collected. Subsequently, protein samples are mixed with loading buffer (Beyotime, Cat.P0015F), denatured by heating (10 minutes at 95° C), and loaded onto 10% sodium dodecyl sulfate-polyacrylamide gel electrophoresis gels (EpiZyme, Cat.PG112) after determining protein concentration using a BCA assay kit (Beyotime, Cat.P0010) for sample quantification. The gel is then run at a constant voltage (120V) until the dye front reaches the bottom. Following electrophoresis, proteins are transferred from the gel to a PVDF using a wet transfer apparatus. The membrane is then blocked with a blocking buffer (CST, Cat.9999S) to prevent nonspecific binding and incubated with primary antibody (1:1000) overnight at 4° C. After washing to remove unbound primary antibody, the membrane is incubated with a secondary antibody (1:2000) conjugated to horseradish peroxidase. Following another round of washing, protein bands are detected using chemiluminescent substrate (EpiZyme, Cat.SQ201) and imaged using a chemiluminescence detection system. JAK3 Antibody (Affbiotech, Cat.AF0008), SUSD4 Antibody (Bioss, Cat.bs-7330R). GAPDH Antibody (CST, Cat.2118S). Secondary Antibody (ZSBiO, Cat.ZB5301).

### Statistical analysis

Statistical analysis was carried out using R 4.2.0 software and the above-mentioned R packages. RNA expression data were log2 (TPM+1) transformed. p < 0.05 was considered statistically different. All experiments were performed in triplicate in at least three separate experiments.

For comparisons between two groups, non-parametric tests or t-tests are employed based on whether the data adhere to a normal distribution. For correlation analysis, the Pearson method is utilized for testing.

## Supplementary Material

Supplementary Figures

Supplementary Tables
