# Supplementary material for: Pan-cancer analysis of Sushi domain-containing protein 4 (SUSD4) and validated in colorectal cancer
Source: Aging (Albany NY). 2024 Apr 4;16(7):6417–44. doi: 10.18632/aging.205712 (PMC11042942; doi:10.18632/aging.205712)
Supplement: Supplementary Figures [file aging-16-205712-s001.pdf]

SUPPLEMENTARY FIGURES

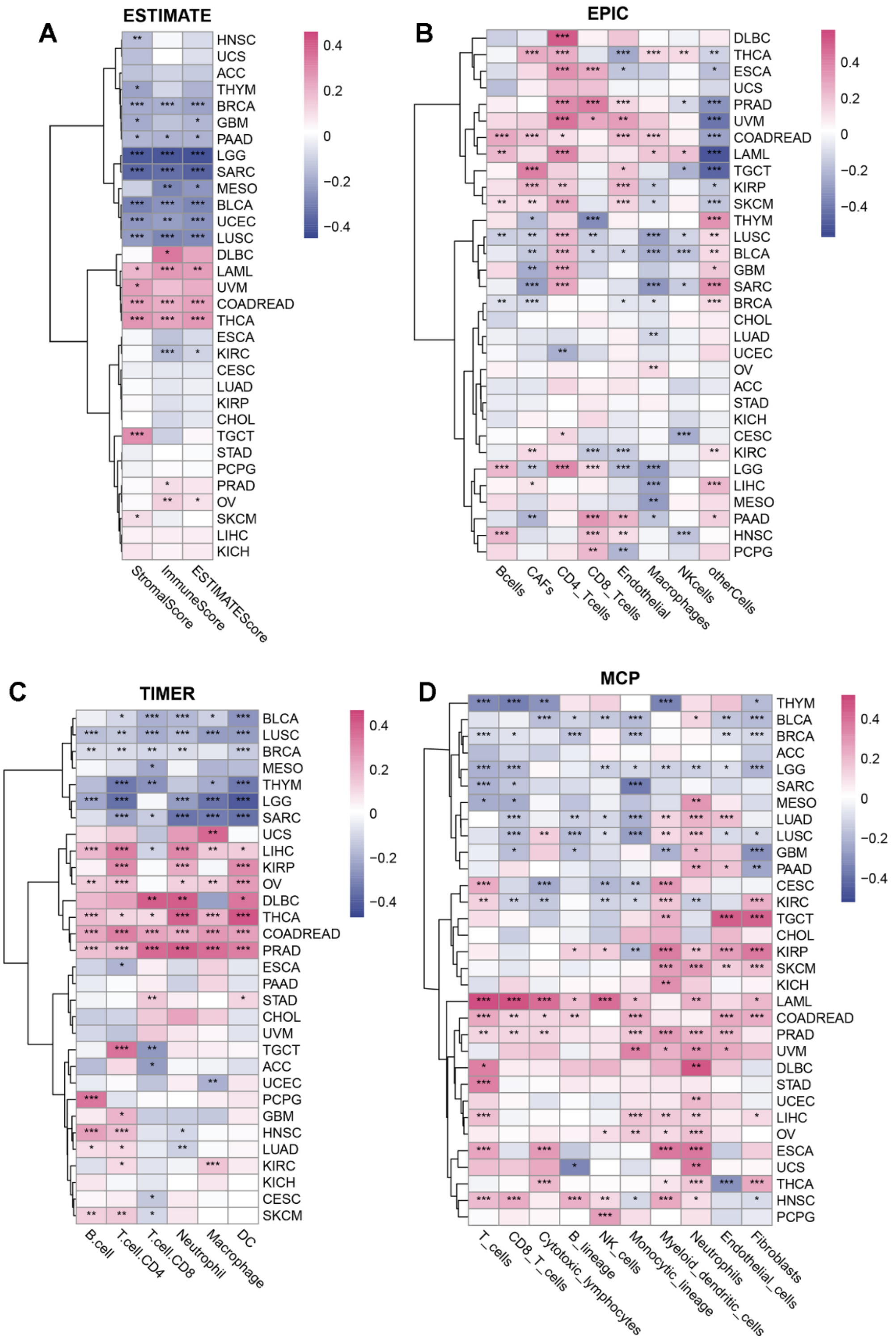

Supplementary Figure 1. The relationship between SUSD4 and immune infiltration. Correlation Analysis Heatmap of SUSD4 with (**A**) ESTIMATE Score, (**B**) EPIC Immune Infiltration, (**C**) Timer Immune Infiltration, and (**D**) MCP Immune Infiltration.

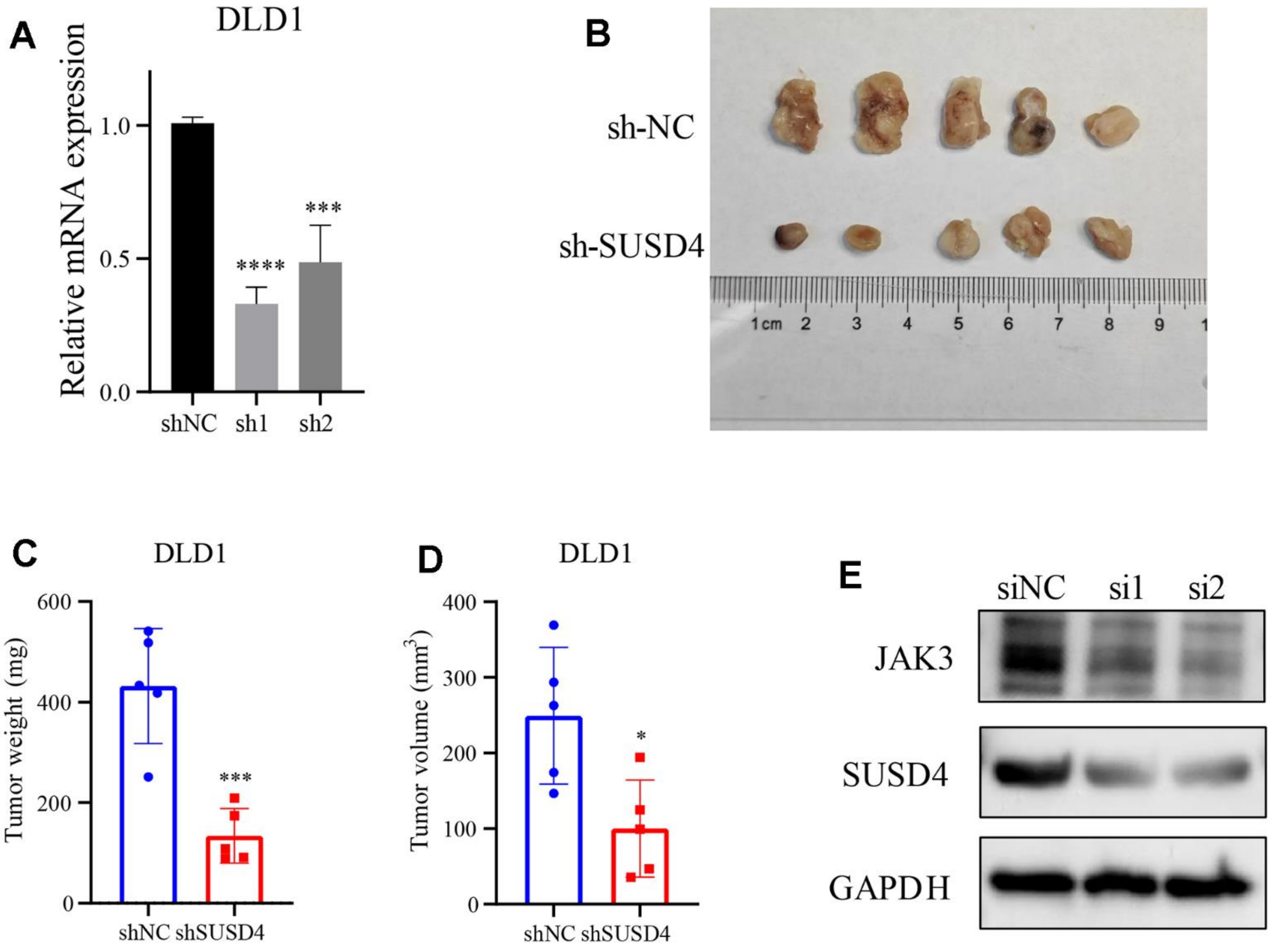

Supplementary Figure 2. (**A**) Confirmation of shRNA knockdown efficiency in DLD1 cells. (**B**) Representative tumor images of DLD1 cells following shSUSD4 and control group treatments. (**C**, **D**) Boxplots illustrating tumor weight (mg) and tumor volume (mm3). (**E**) Western blot analysis depicting the expression levels of JAK3, SUSD4, and GAPDH post siSUSD4 treatment in DLD1 cells. *p<0.05, ***p<0.001, ****p<0.0001.
