# Supplementary material for: Pan-cancer analysis of Sushi domain-containing protein 4 (SUSD4) and validated in colorectal cancer
Source: Aging (Albany NY). 2024 Apr 4;16(7):6417–44. doi: 10.18632/aging.205712 (PMC11042942; doi:10.18632/aging.205712)
Supplement: Supplementary Tables [file aging-16-205712-s002.pdf]

SUPPLEMENTARY TABLES

Supplementary Table 1. Cancer abbreviations.

Abbreviations	Cancer name
ACC	Adrenocortical carcinoma
BLCA	Bladder Urothelial Carcinoma
BRCA	Breast invasive carcinoma
CESC	Cervical squamous cell carcinoma and endocervical adenocarcinoma
CHOL	Cholangiocarcinoma
COAD	Colon adenocarcinoma
COADREAD/COREAD	Colon adenocarcinoma/Rectum adenocarcinoma Esophageal carcinoma
DLBC	Lymphoid Neoplasm Diffuse Large B-cell Lymphoma
ESCA	Esophageal carcinoma
GBM	Glioblastoma multiforme
GBMLGG	Glioma
HNSC	Head and Neck squamous cell carcinoma
KICH	Kidney Chromophobe
KIPAN	Pan-kidney cohort (KICH+KIRC+KIRP)
KIRC	Kidney renal clear cell carcinoma
KIRP	Kidney renal papillary cell carcinoma
LAML	Acute Myeloid Leukemia
LGG	Brain Lower Grade Glioma
LIHC	Liver hepatocellular carcinoma
LUAD	Lung adenocarcinoma
LUSC	Lung squamous cell carcinoma
MESO	Mesothelioma
OV	Ovarian serous cystadenocarcinoma
PAAD	Pancreatic adenocarcinoma
PCPG	Pheochromocytoma and Paraganglioma
PRAD	Prostate adenocarcinoma
READ	Rectum adenocarcinoma
SARC	Sarcoma
STAD	Stomach adenocarcinoma
SKCM	Skin Cutaneous Melanoma
STES	Stomach and Esophageal carcinoma
TGCT	Testicular Germ Cell Tumors
THCA	Thyroid carcinoma
THYM	Thymoma
UCEC	Uterine Corpus Endometrial Carcinoma
UCS	Uterine Carcinosarcoma
UVM	Uveal Melanoma
OS	Osteosarcoma
ALL	Acute Lymphoblastic Leukemia
NB	Neuroblastoma
WT	High-Risk Wilms Tumor

Supplementary Table 2. Primer information.

Gene	Primer-F	Primer-R
**JAK1**	CTTTGCCCTGTATGACGAGAAC	ACCTCATCCGGTAGTGGAGC
**JAK2**	TCTGGGGAGTATGTTGCAGAA	AGACATGGTTGGGTGGATACC
**JAK3**	CCTGATCGTGGTCCAGAGAG	GCAGGGATCTTGTGAAATGTCAT
**STAT1**	CAGCTTGACTCAAAATTCCTGGA	TGAAGATTACGCTTGCTTTTCCT
**STAT2**	GAGCCAGCAACATGAGATTGA	GCCTGGATCTTATATCGGAAGCA
**STAT3**	CAGCAGCTTGACACACGGTA	AAACACCAAAGTGGCATGTGA
**STAT4**	TGTTGGCCCAATGGATTGAAA	GGAAACACGACCTAACTGTTCAT
**STAT5A**	CGACGGGACCTTCTTGTTG	GTTCCGGGGAGTCAAACTTCC
**STAT5B**	GAACACCCGCAATGATTACAGT	ACGGTCTGACCTCTTAATTCGT
**STAT6**	CGAGTAGGGGAGATCCACCTT	GCAGGAGTTTCTATCAAGCTGTG
**SUSD4**	AATGAACCCGAGCAATGGAGA	GGGCTACAGAGCCTTCAAAGA
**GAPDH**	CAGGAGGCATTGCTGATGAT	GAAGGCTGGGGCTCATTT
